# Supplementary material for: Simultaneous Determination of Protein Structure and Dynamics Using Cryo-Electron Microscopy
Source: Biophys J. 2018 Apr 10;114(7):1604–13. doi: 10.1016/j.bpj.2018.02.028 (PMC5954442; doi:10.1016/j.bpj.2018.02.028)
Supplement: Document S1. Supporting Materials and Methods, Figs. S1–S10, and Tables S1 and S2 [file mmc1.pdf]

Biophysical Journal, Volume *114*

Supplemental Information

Simultaneous Determination of Protein Structure and Dynamics Using Cryo-Electron Microscopy

Massimiliano Bonomi, Riccardo Pellarin, and Michele Vendruscolo

Divide-and-conquer GMM fit of cryo-EM maps. To efficiently and accurately fit a high-resolution density map Ψ_D using a GMM with a large number of Gaussians, we used the divide-and-conquer approach developed in Ref. (1). We started from a low-resolution fit of Ψ_D using a GMM with a small number of Gaussians, obtained by an Expectation Maximization algorithm (2). For each component $\phi_{D,i}^1$ of this initial GMM, we defined a submap of the original map

$$\Psi_{D,i}^1(x) = \Psi_D(x) \cdot \frac{\phi_{D,i}^1(x)}{\sum_{j=1}^{N_D^1} \phi_{D,j}^1(x)}$$

Each submap is localized in a subregion where the component $\phi_{D,i}^1$ is localized and the sum all submaps regenerates the original map Ψ_D. The process is repeated and each submap $\Psi_{D,i}^1$ fit using another GMM with small number of Gaussians. At each iteration, the portion of the original map fit by a given GMM becomes smaller and smaller, so that eventually few Gaussians will be sufficient to accurately reproduce high-resolution, local details. By construction, the GMM defined by the union of all the GMMs obtained at a given iteration fits the original map. This approach can be efficiently run in parallel on a cluster until the global GMM reaches the desired accuracy, measured here in terms of cross-correlation with the original map.

For GroEL, we progressively fit the synthetic map using 20, 400, and 4000 Gaussians (**Figure S1**), until reaching a final cross-correlation of over 0.99. In the case of the STRA6 receptors, we fit the experimental map with 20, 4000, and 11585 Gaussians (**Figure S2**), with a final cross-correlation of over 0.97.

Derivation of the forward model. To quantify the agreement of an ensemble of models with the experimental map, we need a forward model, *i.e.* a predictor of the cryo-EM density map from a single structural model. Our forward model is a GMM ϕ_M with N_M Gaussian components. Since here we employed high-resolution synthetic and real cryo-EM maps, we used one component for each heavy atom of the system:

$$\phi_M(\boldsymbol{x}) = \sum_{i=1}^{N_M} \phi_{M,i}(\boldsymbol{x}) = \sum_{i=1}^{N_M} \omega_{M,i} \cdot G\left(\boldsymbol{x} |\, \boldsymbol{x}_{M,i}, \Sigma_{M,i}\right)$$

To derive the parameters of the Gaussian for a given atomic specie (weight and covariance matrix), we fit the tabulated electron scattering form factors (3) for the neutral atom i using a single Gaussian: $f(s) = A_i \exp(-B_i\, s^2)$.

The fitting procedure followed the protocol described in Ref. (4) to fit electron atomic scattering factors with multiple Gaussians. Naturally, the one-Gaussian approximation of the form factor is accurate up to a certain value of s. For density maps of resolution up to ~3 Å, we estimated a maximum relative deviation between tabulated and fitted form factors equal to 1%. In Tab. S1, we report, for neutral C, N, O, and S atoms, the results of the fitting procedure, the range of validity of the one-Gaussian approximation, and the relative maximum error.

From the Gaussian fit of the form factors, we can derive the parameters of the Gaussian in real space (our forward model) by Fourier Transform

$$f(r) = A_i \left(\frac{\pi}{B_i}\right)^{3/2} \exp\left(-\frac{\pi^2}{B_i}\, r^2\right)$$

which leads to the following identities

$$\omega_{M,i} = A_i, \quad \sigma_i = \frac{1}{\pi}\sqrt{\frac{B_i}{2}}, \quad \Sigma_{M,i} = \begin{pmatrix} \sigma_i^2 & 0 & 0 \\ 0 & \sigma_i^2 & 0 \\ 0 & 0 & \sigma_i^2 \end{pmatrix}$$

Enhanced sampling of the metainference ensemble. To accelerate sampling of the metainference ensemble, we used the well-tempered metadynamics algorithm (5). We added an auxiliary variable β to the metainference energy function:

$$E_{MI} = E_{MD} - \frac{k_B T}{\beta}\sum_{r,i} \log\left[\frac{1}{2\left(ov_{DD,i} - \overline{ov}_{MD,i}\right)} \operatorname{erf}\left(\frac{ov_{DD,i} - \overline{ov}_{MD,i}}{\sqrt{2}\,\sigma_{r,i}^{SEM}}\right)\right]$$

with $\beta \geq 1$. The effect of this variable is to weaken the strength of the restraint on the experimental data and avoid the system to get trapped in local free-energy minima. This parameter was sampled using a Monte Carlo (MC) algorithm at every MD simulation step. We defined β on a discrete grid of N_β = 20 bins in the range from β_{min} = 1 to β_{max} = 1000 and distributed it according to

$$\beta_j = \beta_{min} \cdot \exp\left[\frac{j}{N_\beta - 1} \cdot \log\left(\frac{\beta_{max}}{\beta_{min}}\right)\right]$$

with $0 \leq j \leq N_\beta - 1$. For $j = 0$, we recovered the standard metainference score. To accelerate sampling in the β variable, we used a well-tempered metadynamics bias potential V_j constructed by adding at every MC step a "Gaussian" with height equal to (5)

$$\mathrm{W}_0 \cdot \exp\left[-\frac{V_j}{k_B T\,(\gamma - 1)}\right]$$

where W_0 is the initial height and γ the bias factor. The values of W_0 and γ were optimized separately for GroEL and STRA6 simulations. The effect of the bias potential is to ensure efficient diffusion in the space of the β index, which otherwise would be hampered by high free-energy barriers (**Figure S9**). We considered for post-processing all the conformations sampled at $\beta = 1$, as these correspond to the members of the actual metainference ensemble. No further reweighting of these conformations was needed, as the well-tempered metadynamics bias potential tends to become quasi-stationary in the long-time limit and thus all conformations at $\beta = 1$ are sampled under the effect of the same bias potential.

Noise marginalization. We used a Gaussian model of noise with one uncertainty parameter per data point, *i.e.* per component of the data GMM:

$$p\left(ov_{DD,i}|\boldsymbol{X},\sigma_{r,i}\right) = \frac{1}{\sqrt{2\pi}\,\sigma_{r,i}} \cdot \exp\left[-\frac{\left(ov_{DD,i} - \overline{ov}_{MD,i}\right)^2}{2\,\sigma_{r,i}^2}\right]$$

where $ov_{DD,i}$ is the overlap of the i-th component of the data GMM with the entire data GMM. The noise parameter $\sigma_{r,i} = \sqrt{\left(\sigma_{r,i}^{B}\right)^2 + \left(\sigma_{r,i}^{SEM}\right)^2}$ includes all sources of errors (6): errors in the data and forward model $\left(\sigma_{r,i}^{B}\right)$ and the statistical error due to the finite size of the metainference ensemble $\left(\sigma_{r,i}^{SEM}\right)$. This distribution accounts for a variable level of errors across the map, for example due to higher radiation damages to the periphery of the complex. However, sampling all the uncertainty parameters $\sigma_{r,i}$ becomes a daunting task, as high-resolution maps require GMMs with thousands of components. Therefore, we marginalized all the $\sigma_{r,i}$ parameters by integrating the likelihood in combination with a Jeffreys prior $p\left(\sigma_{r,i}\right) = 1/\sigma_{r,i}$, in a range corresponding to

absence of noise in the data ($\sigma_{r,i}^{B} = 0$) to infinite noise ($\sigma_{r,i}^{B} = \infty$). The resulting marginal likelihood is

$$p\left(ov_{DD,i} \mid \boldsymbol{X}\right) = \int_{\sigma_{r,i}^{SEM}}^{\infty} p\left(ov_{DD,i} \middle| \boldsymbol{X}, \sigma_{r,i}\right) p\left(\sigma_{r,i}\right) d\sigma_{r,i}$$

$$= \frac{1}{2\left(ov_{DD,i} - \overline{ov}_{MD,i}\right)} \operatorname{erf}\left(\frac{ov_{DD,i} - \overline{ov}_{MD,i}}{\sqrt{2}\, \sigma_{r,i}^{SEM}}\right)$$

where the error function $\operatorname{erf}(x)$ is defined as

$$\operatorname{erf}(x) = \frac{2}{\sqrt{\pi}} \int_{0}^{x} \exp(-t^2)\, dt$$

The metainference structural ensemble resulting from sampling this marginal posterior is identical to the one that we would obtain by sampling the non-marginal version. However, upon marginalization we lose direct information about the noise level of each region of the map. In the following section, we introduce two approaches to recover *a posteriori* the local level of noise.

Noise inference. In principle, one can use a reweighting procedure to calculate the average value of $\sigma_{r,i}$ for each component of the data GMM. $p\left(\sigma_{r,i} \middle| \phi_D\right)$ can be estimated from a sample of the metainference posterior $p(\boldsymbol{X}|\phi_D)$ in the following way. We start by noting that

$$p\left(\sigma_{r,i} \middle| \phi_D\right) = \int d\boldsymbol{X} \int d\boldsymbol{\sigma}\ p(\boldsymbol{X}, \boldsymbol{\sigma}|\phi_D)$$

where the integral in $\boldsymbol{\sigma}$ is over all the $\sigma_{s,j}$ with $s \neq r$ or $j \neq i$. We can multiply and divide the integrand by $p(\boldsymbol{X}|\phi_D)$

$$p(\sigma_{r,i}|\phi_D) = \int d\boldsymbol{X} \int d\boldsymbol{\sigma} \, \frac{p(\boldsymbol{X},\boldsymbol{\sigma}|\phi_D)}{p(\boldsymbol{X}|\phi_D)} \cdot \, p(\boldsymbol{X}|\phi_D) = \langle \int d\boldsymbol{\sigma} \, \frac{p(\boldsymbol{X},\boldsymbol{\sigma}|\phi_D)}{p(\boldsymbol{X}|\phi_D)} \rangle_{MI}$$

where the average $\langle\cdot\rangle$ is taken over the metainference simulations. If carry out the integration in $\boldsymbol{\sigma}$ at the numerator, we obtain

$$p(\sigma_{r,i}|\phi_D) = \langle \frac{p(\phi_{D,i}|\boldsymbol{X},\sigma_{r,i}) \cdot p(\sigma_{r,i})}{p(\phi_{D,i}|\boldsymbol{X})} \rangle_{MI}$$

which allows to numerically estimate $p(\sigma_{r,i}|\phi_D)$ from the average of known quantities calculated *a posteriori* over the metainference simulations. The average error is then calculated as $\langle\sigma_{r,i}\rangle = \int \sigma_{r,i} \; p(\sigma_{r,i}|\phi_D) \, d\sigma_{r,i}$.

Alternatively, one can infer the most probable local level of noise from the entire ensemble $\boldsymbol{X}$ generated by the metainference simulations. $\boldsymbol{X}$ contains all conformations generated by all replicas during the metainference run. For each component of the data GMM, the probability of having a noise level equal to σ_i, given the ensemble and the data is:

$$p(\sigma_i|\boldsymbol{X},\phi_{D,i}) = \frac{p(\boldsymbol{X},\sigma_i|\phi_{D,i})}{p(\boldsymbol{X}|\phi_{D,i})} \propto p(\phi_{D,i}|\boldsymbol{X},\sigma_i) \cdot p(\sigma_i)$$

where we omitted all terms independent from the level of noise, as these are constant in this post-processing stage. If we use the same Gaussian noise model and Jeffreys prior for σ_i employed in the generation of models, we obtain:

$$p(\sigma_i|\boldsymbol{X},\phi_{D,i}) \propto \frac{1}{\sigma_i^2} \cdot \exp\left[-\frac{\left(ov_{DD,i} - \overline{ov}_{MD,i}\right)^2}{2\,\sigma_i^2}\right]$$

where $\overline{ov}_{MD,i}$ is the average overlap calculated over the entire metainference ensemble $\boldsymbol{X}$.

From this relation, we obtain the probability of the relative noise level $\sigma_i^{rel} = \sigma_i/ov_{DD,i}$ from a simple change of variable:

$$p\left(\sigma_i^{rel}|\boldsymbol{X},\phi_{D,i}\right) \propto \frac{1}{\left(\sigma_i^{rel}\right)^2}\cdot \exp\left[-\frac{\left(ov_{DD,i}-\overline{ov}_{MD,i}\right)^2}{2\,ov_{DD,i}^2\left(\sigma_i^{rel}\right)^2}\right] = \frac{1}{\left(\sigma_i^{rel}\right)^2}\cdot \exp\left[-\frac{\Delta_i^2}{2\left(\sigma_i^{rel}\right)^2}\right]$$

Δ_i is the relative deviation of the experiment from the prediction and can be back-calculated, for each compoenent of the data GMM, *a posteriori* from the ensemble $\boldsymbol{X}$. At this point, the most likely level of relative noise is defined as the value $\overline{\sigma_i^{rel}}$ that maximizes $p\left(\sigma_i^{rel}|\boldsymbol{X},\phi_{D,i}\right)$:

$$\overline{\sigma_i^{rel}} = \frac{\Delta_i}{\sqrt{2}}$$

In this work, we adopted this simpler approach to calculate the error map for GroEL and STRA6 (**Figures 1E, 2E, and S4E**), following the procedure described in the next section.

Noise map. To visualize the relative error $\overline{\sigma_i^{rel}}$ associated to each component of the data GMM ϕ_D along with the experimental map, we first created a voxel-representation of ϕ_D using the *gmconvert* utility (2). We then defined an error map σ_D on the same grid as ϕ_D

$$\sigma_D(\boldsymbol{x}) = \frac{\sum_{i=1}^{N_D}\overline{\sigma_i^{rel}}\cdot\phi_{D,i}(\boldsymbol{x})}{\sum_{i=1}^{N_D}\phi_{D,i}(\boldsymbol{x})}$$

and used UCSF Chimera (7) to color the voxel-representation of ϕ_D using σ_D (**Figures 1E, 2E, and S4E**).

Supplementary References

1. Hanot, S., M. Bonomi, C. H. Greenberg, A. Sali, M. Nilges, M. Vendruscolo, and R. Pellarin. 2017. Bayesian multi-scale modeling of macromolecular structures based on cryo-electron microscopy density maps. bioRxiv doi: 10.1101/113951.
2. Kawabata, T. 2008. Multiple Subunit Fitting into a Low-Resolution Density Map of a Macromolecular Complex Using a Gaussian Mixture Model. Bioph. J. 95:4643-4658.
3. Prince, E. 2004. International Tables for Crystallography Vol. C. Wiley, Hoboken.
4. Peng, L. M., G. Ren, S. L. Dudarev, and M. J. Whelan. 1996. Robust parameterization of elastic and absorptive electron atomic scattering factors. Acta Crystallogr. A 52:257-276.
5. Barducci, A., G. Bussi, and M. Parrinello. 2008. Well-tempered metadynamics: A smoothly converging and tunable free-energy method. Phys. Rev. Lett. 100.
6. Bonomi, M., C. Camilloni, A. Cavalli, and M. Vendruscolo. 2016. Metainference: A Bayesian inference method for heterogeneous systems. Sci. Adv. 2:e1501177.
7. Pettersen, E. F., T. D. Goddard, C. C. Huang, G. S. Couch, D. M. Greenblatt, E. C. Meng, and T. E. Ferrin. 2004. UCSF Chimera--a visualization system for exploratory research and analysis. J. Comp. Chem. 25:1605-1612.
8. Laskowski, R. A., M. W. Macarthur, D. S. Moss, and J. M. Thornton. 1993. Procheck - a Program to Check the Stereochemical Quality of Protein Structures. J. Appl. Crystallogr. 26:283-291.

Supplementary Tables

Table S1. Summary of the building blocks of the metainference approach to model structure and dynamics from cryo-EM data.

#	Name	Equation	Notes	PLUMED keywords
1	normalized Gaussian function	$G(\boldsymbol{x}\vert\, \overline{\boldsymbol{x}},\Sigma) = \frac{1}{(2\pi)^{\frac{3}{2}}\vert\Sigma\vert^{\frac{1}{2}}}\exp\left[-\frac{1}{2}(\boldsymbol{x}-\overline{\boldsymbol{x}})^T(\Sigma)^{-1}(\boldsymbol{x}-\overline{\boldsymbol{x}})\right]$		
2	j-th component of model-GMM	$\phi_{M,j}(\boldsymbol{x}) = \omega_{M,j}\cdot G\left(\boldsymbol{x}\mid \boldsymbol{x}_{M,j},\Sigma_{M,j}\right)$	differentiable function of the model coordinates	
3	i-th component of data-GMM	$\phi_{D,i}(\boldsymbol{x}) = \omega_{D,i}\cdot G\left(\boldsymbol{x}\mid \boldsymbol{x}_{D,i},\Sigma_{D,i}\right)$		
4	model-GMM	$\phi_M(\boldsymbol{x}) = \sum_{j=1}^{N_M}\phi_{M,j}(\boldsymbol{x})$	forward model to predict a density map from the model	
5	data-GMM	$\phi_D(\boldsymbol{x}) = \sum_{i=1}^{N_D}\phi_{D,i}(\boldsymbol{x})$	GMM fit of the experimental map	GMM_FILE
6	overlap of two GMM components	$ov_{M,j\;D,i} = \int d\boldsymbol{x}\;\phi_{M,j}(\boldsymbol{x})\,\phi_{D,i}(\boldsymbol{x}) = \frac{\omega_{M,j}\,\omega_{D,i}}{(2\pi)^{3/2}\vert\Sigma_{M,j}+\Sigma_{D,i}\vert^{1/2}}\exp\left[-\frac{1}{2}\left(\boldsymbol{x}_{M,j}-\boldsymbol{x}_{D,i}\right)^T\left(\Sigma_{M,j}+\Sigma_{D,i}\right)^{-1}\left(\boldsymbol{x}_{M,j}-\boldsymbol{x}_{D,i}\right)\right]$	overlap of the j-th component of model-GMM with the i-th component of data-GMM	
7	total overlap	$ov_{MD,i} = \int d\boldsymbol{x}\;\phi_M(\boldsymbol{x})\,\phi_{D,i}(\boldsymbol{x}) = \sum_{j=1}^{N_M} ov_{M,j\;D,i}$	total overlap between model-GMM and i-th component of data-GMM	NL_CUTOFF NL_STRIDE

8	average total overlap	$\int d\boldsymbol{x} \left(\frac{1}{N} \sum_{r=1}^{N} \phi_M^r(\boldsymbol{x}) \right) \phi_{D,i}(\boldsymbol{x}) = \frac{1}{N} \sum_{r=1}^{N} ov_{MD,i}^r = \overline{ov}_{MD,i}$	total overlap of model-GMM averaged across the metainference replicas	
9	experimental overlap	$ov_{DD,i} = \int d\boldsymbol{x}\ \phi_D(\boldsymbol{x})\ \phi_{D,i}(\boldsymbol{x})$	total overlap between data-GMM and i-th component of data-GMM	
10	data-restraint for the i-th component of data-GMM	$E_{D,i} = -k_B T \sum_r \log \left[\frac{1}{2\left(ov_{DD,i} - \overline{ov}_{MD,i}\right)} \right] - k_B T \sum_r \log \left[\mathrm{erf} \left(\frac{ov_{DD,i} - \overline{ov}_{MD,i}}{\sqrt{2}\, \sigma_{r,i}^{SEM}} \right) \right]$	Obtained from marginalization of Gaussian noise	SIGMA_MEAN TEMP
11	total data-restraint	$E_{D=} \sum_{i=1}^{N_D} E_{D,i}$	sum over all the components of the data-GMM	EMMI
12	metainference energy function	$E_{MI} = E_{MD} + E_D$		

Table S2. Parameters of the forward model. The electron atomic scattering factors for C, N, O, and S neutral atoms were fit using a single Gaussian function $f(s) = A_i \exp(-B_i\, s^2)$. For each atom, we report the best fit of the A and B coefficients, the maximum value of s used in the fitting procedure (s_{max}), the lower bound in resolution for the validity of the single-Gaussian approximation (d_{min}), and the maximum error (err_{max}), defined as maximum relative deviation of the fit from the tabulated atomic scattering factor in the range $0 \leq s \leq s_{max}$.

Atom type	A	B [Å^2]	s_{max} [1/Å]	d_{min} [Å]	err_{max}
C	2.50	15.15	0.15	3.3	0.0101
N	2.20	11.11	0.17	2.9	0.0095
O	1.98	8.60	0.19	2.6	0.0093
S	5.14	15.90	0.15	3.3	0.0109

Supplementary Figures

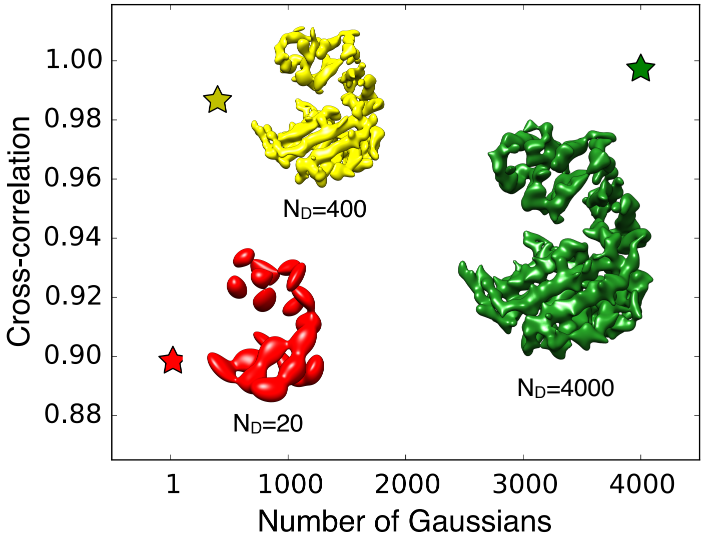

Figure S1. Cross-correlation of the GMM fit with the synthetic GroEL map as a function of the number of GMM components. The cross correlation was 0.90 for 20 Gaussian components (red star), 0.985 for 400 Gaussian components (yellow star), and 0.995 for 4000 Gaussian components (green star).

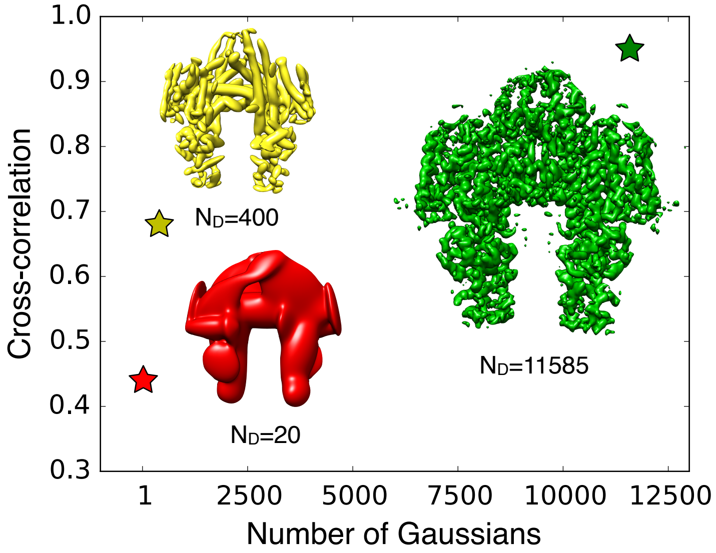

Figure S2. Cross-correlation of the GMM fit with the STRA6 experimental map (EMD code 8315) as a function of the number of GMM components. The cross correlation was 0.44 for 20 Gaussian components (red star), 0.68 for 400 Gaussian components (yellow star), and 0.97 for 11585 Gaussian components (green star).

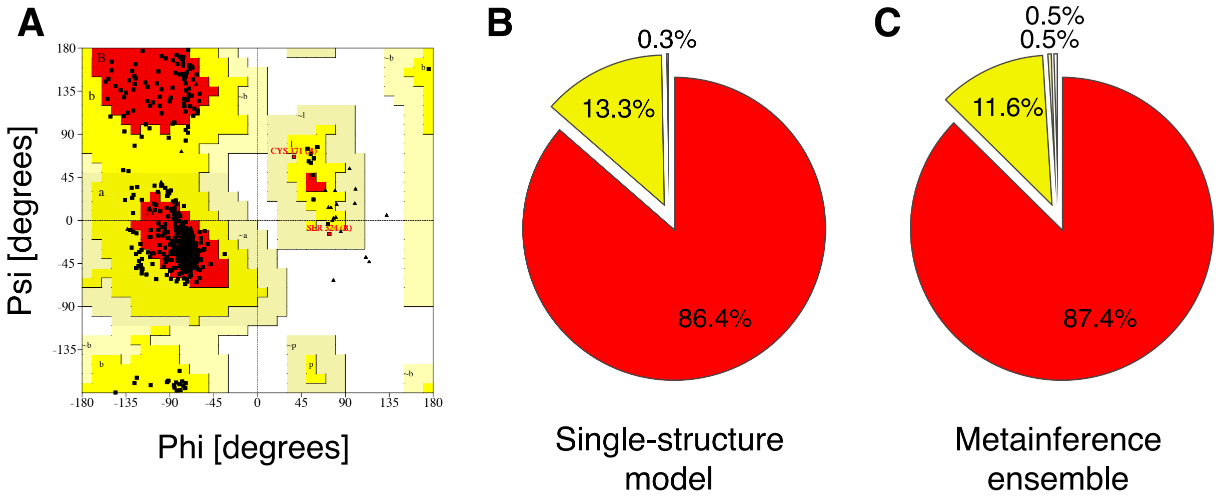

Figure S3. Stereochemistry assessment of the STRA6 single-structure deposited model and metainference ensemble. PROCHECK (8) was used to calculate the distributions of backbone dihedral angles across all residues and models. Dihedrals were then classified in 4 regions of the Ramachandran plot (A): residues in most favoured regions (red), in additional allowed regions (yellow), in generously allowed regions (light yellow), and in disallowed regions (white). The percentages of residues in each of the four regions is reported for the single-structure model (B) and the metainference ensemble (C).

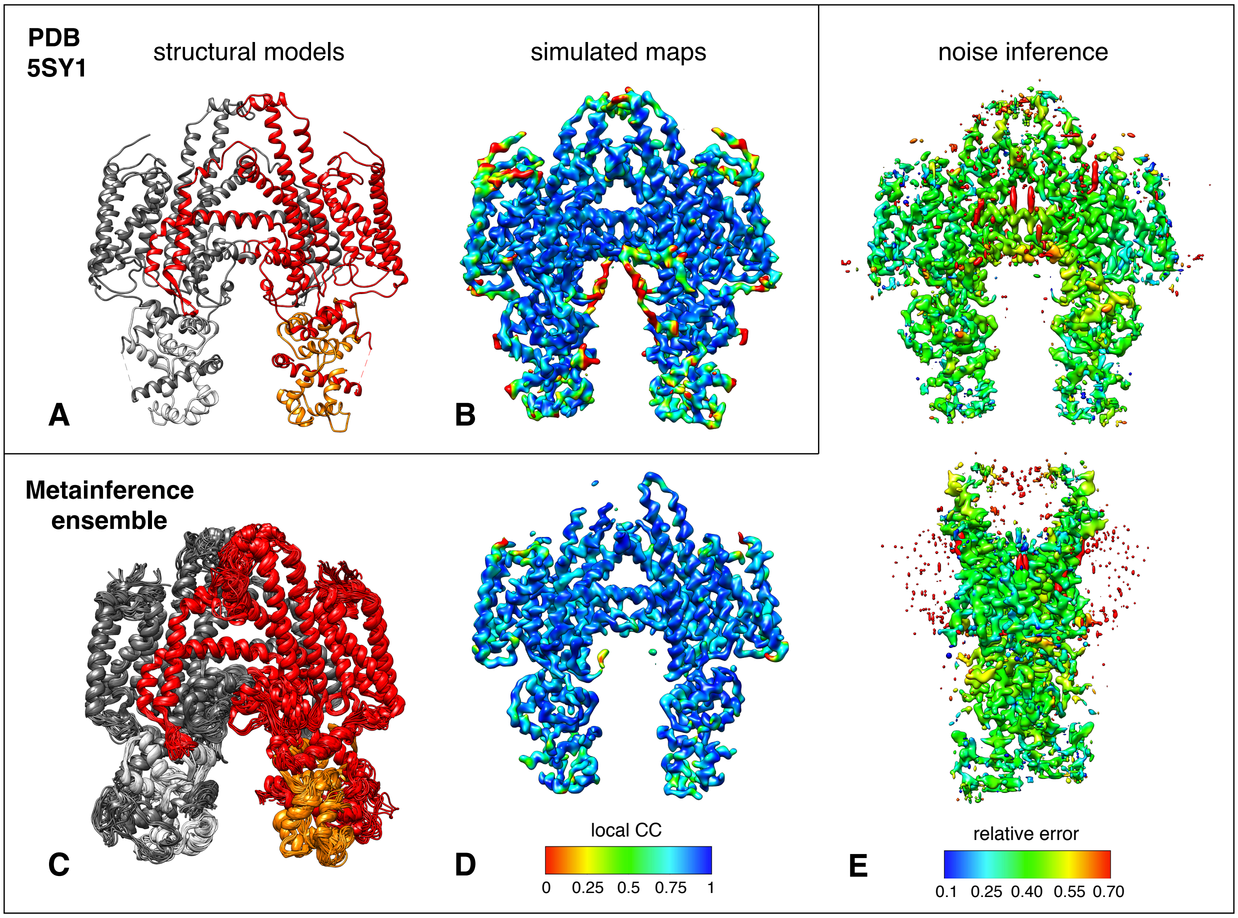

Figure S4. Application of the metainference method to the STRA6 membrane complex. We report the same analysis of **Figure 2** for the second independent metainference run.

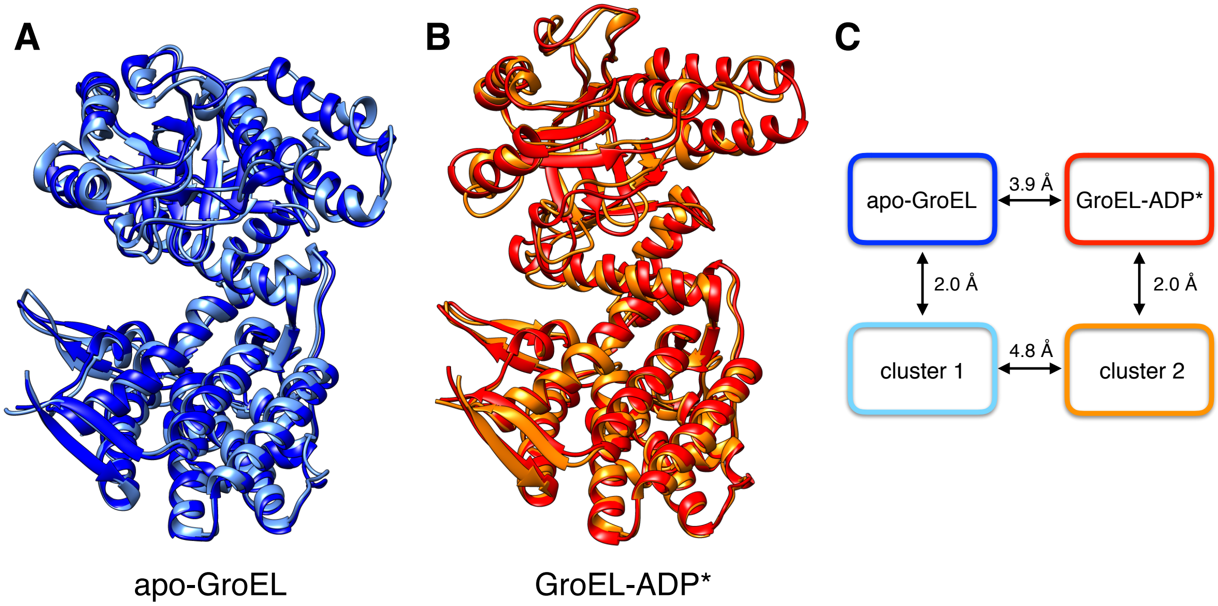

Figure S5. (A) Comparison of the crystal structure of apo GroEL (PDB code 1XCK, blue) with the center of cluster 1 of the metainference simulations (cyan). (B) Comparison of GroEL-ADP* (red), a model built from the extended allosteric state adopted by GroEL in complex with ADP (PDB code 4KI8) with the center of cluster 2 of the metainference simulations (orange). (C) Summary of the backbone RMSD values between input structures and metainference models.

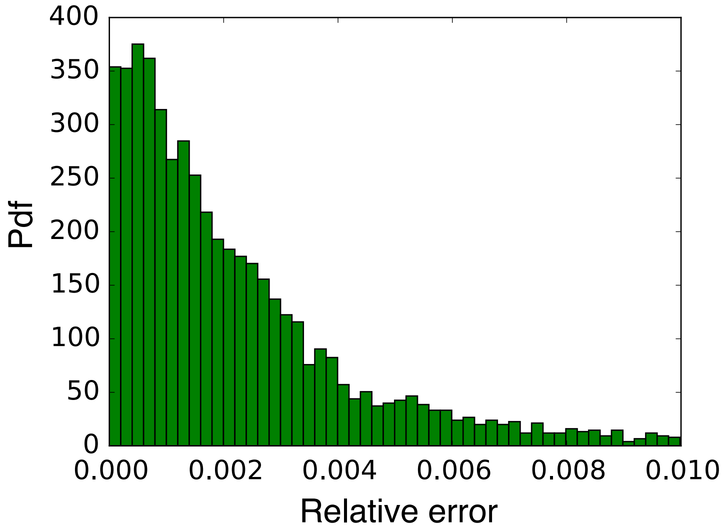

Figure S6. Distribution, in terms of a probability density function (Pdf), of the inferred level of relative noise across all components of the GroEL data GMM.

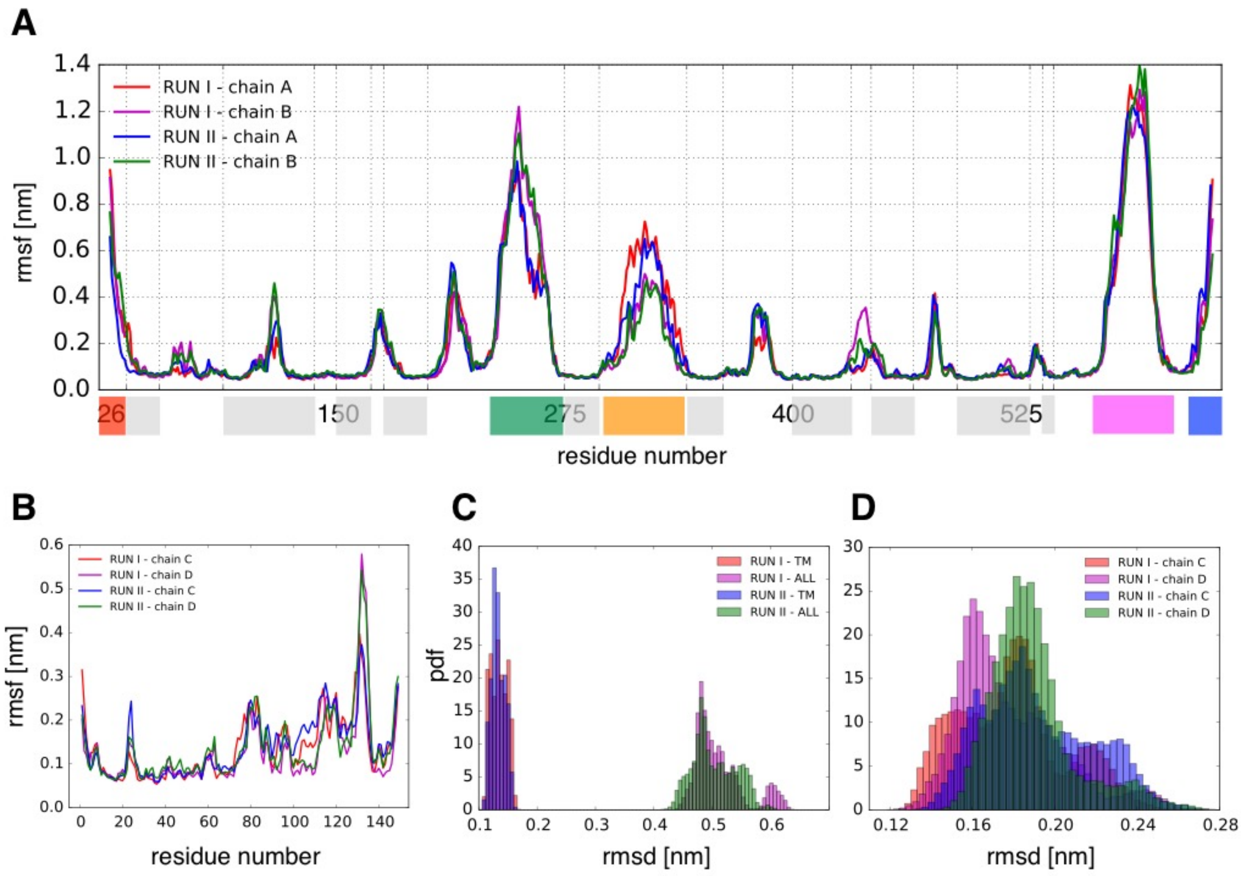

Figure S7. Root-mean-square fluctuation (rmsf) calculated on the Cα atoms of the STRA6 receptor (A), independently for the two identical chains of the dimer and in the two production runs (red, magenta, blue, and green lines). On the x-axis, specific regions of the STRA6 structure are highlighted along the sequence using different colors: the N-terminal domain (red), the TM domain (grey), the JM helix (green), the RBP-binding motif and LP (orange), the cytosolic loop (magenta), and the C-terminal domain (blue). Rmsf calculated on the Cα atoms of the calmodulin domain (B), independently for the two identical chains of the dimer and in the two production runs (red, magenta, blue, and green lines). Distribution of backbone RMSD from the single-deposited model calculated on the TM region (red and blue bars) and on the entire STRA6 receptor (magenta and green bars) for the two metainference production runs (C). In all cases,

prior to RMSD calculations, all conformations were aligned on the atoms belonging to the TM, defined by the region 12.5 nm < z < 15.0 nm in the single-structure deposited model. Distribution of backbone RMSD from the single-deposited model calculated on the calmodulin domain for the two indentical chains of the receptor and in the two production runs (D).

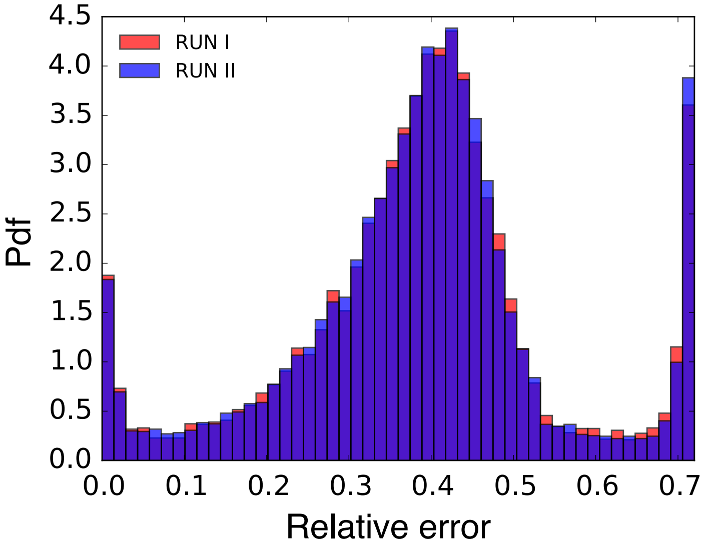

Figure S8. Distributions of the inferred level of relative noise across all components of the STRA6 data GMM in the two independent runs (red and blue bars for RUN I and RUN II, respectively).

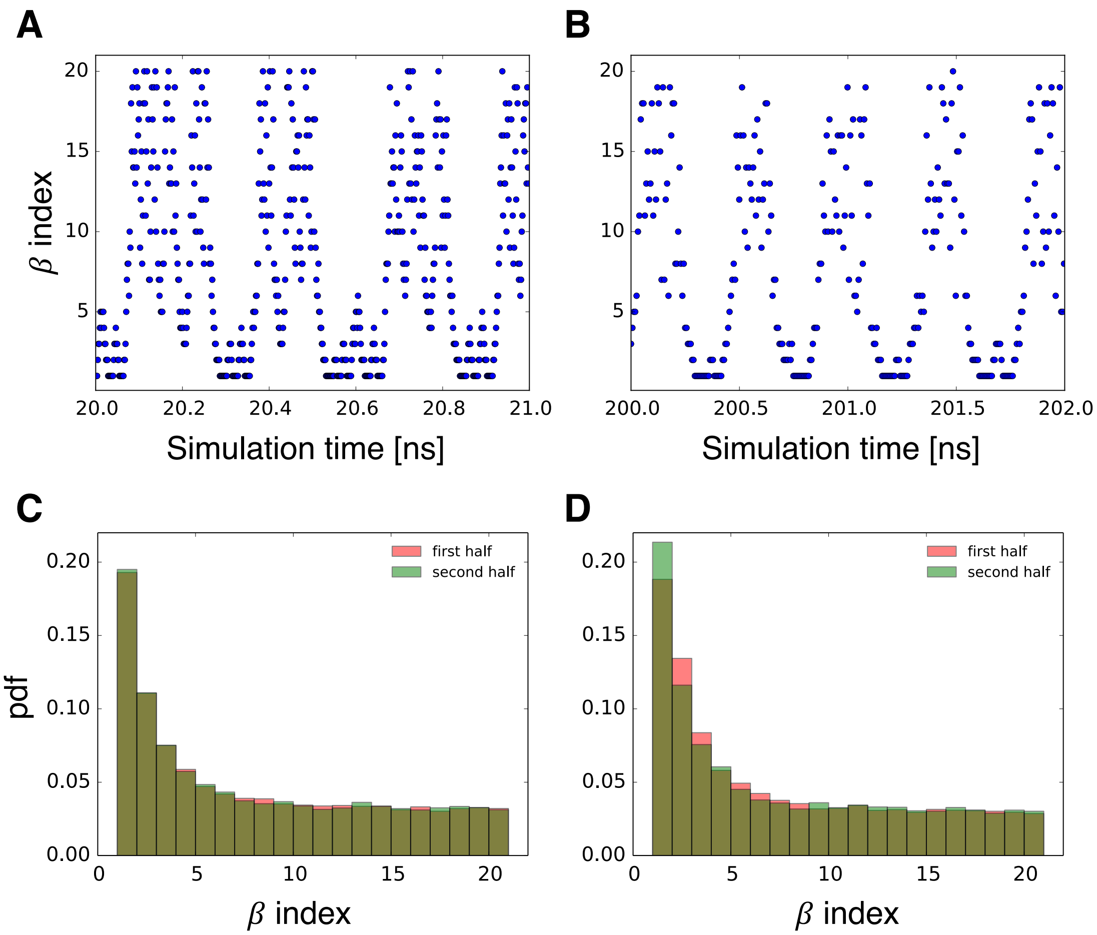

Figure S9. Efficient diffusion in β space during a representative segment of the GroEL (A) and STRA6 (B) metainference simulations. Distributions of the β index calculated over the first (red) and second (green) half of the GroEL (C) and STRA6 (D) simulations.

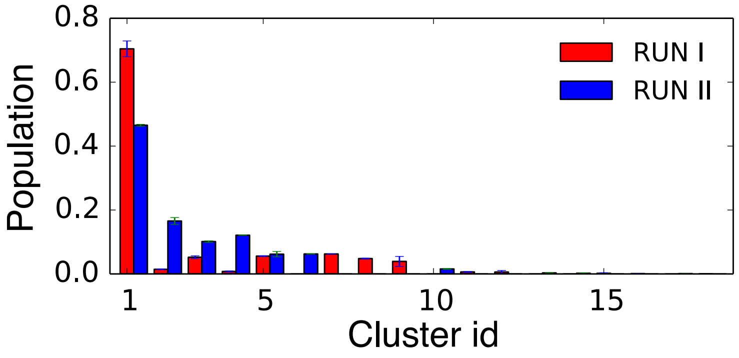

Fig. S10. Convergence assessment of the STRA6 metainference simulations. All conformations generated in the two production runs were clustered together using the GROMOS algorithm, using as metrics the backbone RMSD and a cutoff of 0.35 nm. The average and standard deviation of the populations calculated in the first and second half of each production run are reported (red and blue bars for RUN I and RUN II, respectively).
